# Supplementary material for: Plant-Growth Endophytic Bacteria Improve Nutrient Use Efficiency and Modulate Foliar N-Metabolites in Sugarcane Seedling
Source: Microorganisms. 2021 Feb 25;9(3):479. doi: 10.3390/microorganisms9030479 (PMC7996552; doi:10.3390/microorganisms9030479)
Supplement: Supplementary file 1 [file microorganisms-09-00479-s001.pdf]

Supplementary material

Supplementary Table S1. Soil chemical characterization.

pH	5.5
CEC	120
BS((mg/dm^3)	77
P (mg/dm^3)	23
H + Al (mmolc/dm^3)	28
Base saturation (mmol$_c$dm^{-3})	92
Organic matter (gdm^{-3})	27
K (mmol$_c$dm^{-3})	5
Ca (mmol$_c$dm^{-3})	76
Mg (mmol$_c$dm^{-3})	11
B (mgdm^{-3})	0.24
Cu (mgdm^{-3})	5.2
Fe (mgdm^{-3})	5.2
Mn (mgdm^{-3})	13.9
Zn (mgdm^{-3})	1.6

CEC: cation exchange capacity; BS: basic saturation; SB: sum of bases

Supplementary Table S2. *In vitro* activity of nitrate reductase (NR), nitrite reductase (NiR) and glutamine synthetase (GS) of sugarcane leaves.

Treatments	NR[1]	NiR[1]	GS[2]
Control	11.82 a*	8.04 a	11.85 a
IAC/BECa-088	12.05 a	8.17 a	12.45 a
IAC/BECa-090	11.56 a	8.01 a	13.01 a
IAC/BECa-095	10.94 a	8.30 a	11.57 a
IAC/BECa-135	11.27 a	8.56 a	13.37 a
IAC/BECa-141	11.98 a	8.53 a	12.16 a
IAC/BECa-152	11.66 a	7.94 a	11.52 a

[1]nmol NO_2^- g^{-1} FM h^{-1}; [2]μmol GGH g^{-1}MF h^{-1}.* means followed by similar letters are not significantly by Scott-Knott test ($p <$ 0.05).

Supplementary Table S3. Profile of amino acids and polyamines in sugarcane leaves in function of bacterial treatments, 45 days after transplanting.

Treatments	Putrescine	Lysine	Glycine	Gaba	Alanine	Citrulline	Glutamate	Threonine	Aspartate	Glutamine	Proline	Valine	Phenylalanine
	μmol g^{-1}DW												
Control	880.3 a*	16.83 a	277.5 b	6.58 a	755.9 b	16.97 a	7.87 a	36.17 a	166.4 a	7.23 a	104.22 a	14.57 a	326.5 a
IAC/BECa-088	523.2 b	16.21 a	376.5 b	6.40 a	2150.9 a	16.38 a	8.49 a	37.46 a	195.7 a	2.79 b	99.64 a	14.52 a	344.1 a
IAC/BECa-090	565.2 b	20.15 a	616.1 a	5.11 a	1089.7 b	13.52 b	6.15 a	33.88 a	161.4 a	5.87 a	70.63 b	16.34 a	376.0 a
IAC/BECa-095	635.0 b	16.04 a	485.2 a	6.24 a	2064.3 a	12.45 b	8.34 a	37.80 a	165.0 a	4.29 b	93.32 a	18.02 a	382.2 a
IAC/BECa-135	450.9 b	17.99 a	475.2 a	5.86 a	608.6 b	11.75 b	7.88 a	32.92 a	108.0 b	4.86 a	75.96 b	15.55 a	303.7 a
IAC/BECa-141	564.5 b	13.8 a	397.5 b	4.63 a	1029.9 b	11.52 b	3.23 b	26.08 a	98.9 b	2.36 b	67.44 b	15.63 a	285.0 a
IAC/BECa-152	580.2 b	24.98 a	591.4 a	3.70 a	500.5 b	11.94 b	4.30 b	36.46 a	95.4 b	6.38 a	82.27 b	16.74 a	414.5 a
CV%	34.33	35.3	26.42	38.05	63.97	27.14	44.93	28.23	21.79	54.04	24.97	21.85	24.97

* means followed by similar letters are not significantly by Scott-Knott test ($P<0.05$). CV(%): coefficient of variation.
